# Supplementary material for: Promoting electricity conservation through behavior change: A study protocol for a web-based multiple-arm parallel randomized controlled trial
Source: PLoS One. 2024 Mar 14;19(3):e0293683. doi: 10.1371/journal.pone.0293683 (PMC10939288; doi:10.1371/journal.pone.0293683)
Supplement: S1 Table — (DOCX) [file pone.0293683.s001.docx]

**S1 Table.** *Hypotheses considering main and interaction effects of independent and dependent variables in the rct and expected results*

|  | **Hypotheses** |
| --- | --- |
|  | Information about energy saving tips will reduce the normalized electricity use of the targeted households per week as compared to the baseline and to the control groups |
|  | Information and providing feedback about a household's energy use will reduce the normalized electricity use of the targeted households per week as compared to the baseline, the control groups, and the information alone condition |
|  | Providing information and social norm comparison (other people's electricity consumption) will reduce the normalized electricity use of the targeted households per week as compared to the baseline, the control groups, and the information alone condition for people consuming above average electricity |
|  | Information and a commitment to save 5% of their electricity consumption in the following week will have a higher electricity saving as compared to the baseline, the control groups, and the information alone condition |
|  | Information, commitment to save 5% of their electricity consumption in the following week and have this commitment displayed on the landing page of the platform will have a higher electricity saving as compared to the baseline, the control groups, and the information alone condition and people who commit to electricity saving but do not agree to this commitment being public |
|  | Information and participation in a competition where the own electricity savings are ranked against the electricity saving champions (10 best performing) will increase electricity saving as compared to the baseline, the control groups, and the information alone condition |
|  | Information and framing the electricity saving tips in a collective form (e.g., "we can save ...") will increase electricity saving in comparison to the individual condition ("you can save ...") |
|  | Changes in behavior initiated by the interventions are mediated through intentions, attitudes, perceived behavioral control, social norms, and emotional reactions to the interventions) |
|  | The combination of information, social norm, and collective framing will have a larger electricity saving effect than the individual interventions and the control groups |
|  | The combination of information, feedback, and collective framing will have a larger electricity saving effect than the individual interventions and the control groups |
|  | The combination of information, feedback, and competition will have a larger electricity saving effect than the individual interventions and the control groups |
|  | The combination of information, feedback, competition, and collective framing will have a larger electricity saving effect than the individual interventions and the control groups |
|  | The combination of information, social norms, and commitment will have a larger electricity saving effect than the individual interventions and the control groups |
|  | The combination of information, social norm, commitment, feedback, and competition will have a larger electricity saving effect than the individual interventions and the control groups |
|  | The combination of information, social norm, commitment, feedback, competition, and collective framing will have a larger electricity saving effect than the individual interventions and the control groups |
|  | The effectiveness of the interventions will depend on the country they are implemented in (undirected country differences expected) |
|  | The effectiveness of the interventions will depend on the user partner that communicated to the participants (undirected differences between types of user partners expected) |
|  | The effectiveness of the interventions will depend on the channel people have been invited through (undirected communication |
|  | The effects of collective framing are moderated by identification with the country the participants live in (which is the unit used for the collective framing) and perceived collective efficacy. |
|  | The effect of the interventions is moderated by the habit strength of energy saving behavior. |
