# Supplementary material for: Promoting electricity conservation through behavior change: A study protocol for a web-based multiple-arm parallel randomized controlled trial
Source: PLoS One. 2024 Mar 14;19(3):e0293683. doi: 10.1371/journal.pone.0293683 (PMC10939288; doi:10.1371/journal.pone.0293683)
Supplement: S2 Table — (DOCX) [file pone.0293683.s002.docx]

**S2 Table.** *Items pertaining to electricity-related behaviors/values/habits with substantial potential for energy conservation.*

| **Socio-demographics** [1] | | | | | | | | |
| --- | --- | --- | --- | --- | --- | --- | --- | --- |
| 1 | Which year were you born? |  |  |  |  |  |  |  |
| 2 | Which is your gender? | male | female | other |  |  |  |  |
| 3 | In which County of [your country] do you live? |  |  |  |  |  |  |  |
| 4 | How many people live in your house/apartment? |  |  |  |  |  |  |  |
| 5 | How many of these are under 6 years old? |  |  |  |  |  |  |  |
| 6 | How many of these are between 6 and 11? |  |  |  |  |  |  |  |
| 7 | How many of these are between 12 and 17? |  |  |  |  |  |  |  |
| 8 | What is your highest education level? | basic education | vocational training | highschool degree | university degree | still in education |  |  |
| 9 | What describes best your job situation? | working full time | working part time | in fulltime education | without paid work / looking for work | retired | not able to work | other |
| 10 | How would you describe your social status? | 1 = worst-off |  |  |  |  |  | 10 = best off |
| 11 | Being a citizen of [your country] is an important part of who I am | 1= strongly disagree | 2 = moderately disagree | 3= neither disagree nor agree | 4 = moderately agree | 5 = strongly agree |  |  |
| **Risk of Energy Poverty** | | | | | | | | |
| 11 | Do you struggle to pay for your electricity bill, because it takes too much from your monthly income? | never | rarely | sometimes | often | always |  |  |
| 12 | On average across the year, how much of your household’s income did you use to pay for energy (electricity, wood, gas, oil, gasoline, diesel etc. together) during the last 12 months? | 0 | below 5% | 5-10% | 10-15% | 15-20% | 20-30% | more than 30% |
| **Environmental Concern [2]** | | | | | | | | |
| 13 | How worried are you about climate change? | very worried/concerned | somewhat worried/ concerned | not very worried/concerned | not at all worried/concerned |  |  |  |
| 14 | How concerned are you generally about environmental problems? | very worried/concerned | somewhat worried/ concerned | not very worried/concerned | not at all worried/concerned |  |  |  |
| 15 | How concerned are you generally about using too much electricity? | very worried/concerned | somewhat worried/ concerned | not very worried/concerned | not at all worried/concerned |  |  |  |
| **Personal Norms [3]** | | | | | | | | |
| 16 | Because of my personal values, I feel morally obliged to save electricity | very easy to do | somewhat easy to do | neither easy nor difficult | somewhat difficult to do | very difficult to do | not relevant for me / do not have that |  |
| ***Electricity Assets in the Household (EAH)*** | | | | | | | | |
| 17 | Do you use electricity to warm your hot water? | no | yes | do not know |  |  |  |  |
| 18 | Do you use electricity as a main heating source? | no | yes | do not know |  |  |  |  |
| 19 | Do you charge an electric car at home | no | yes | do not know |  |  |  |  |
| 20 | I have downloaded the app of my electricity net provider or have checked my consumption on their website. | no | yes | do not know |  |  |  |  |
| 21 | I have an air condition | no | yes | do not know |  |  |  |  |
| 22 | I have a tumble dryer | no | yes | do not know |  |  |  |  |
| 23 | I have a heat pump | no | yes | do not know |  |  |  |  |
| 24 | I have a dishwasher which is highly energy efficient | no | yes | do not know |  |  |  |  |
| 25 | I have a washing machine which is highly energy efficient | no | yes | do not know |  |  |  |  |
| 26 | I have LED lights installed everywhere I can | no | yes | do not know |  |  |  |  |
| **Perceived Behavioral Control of electricity-saving tips (PBC)[3]** | | | | | | | | |
| 27 | Keeping lids on pots when cooking | very easy to do | somewhat easy to do | neither easy nor difficult | somewhat difficult to do | very difficult to do | not relevant for me / do not have that |  |
| 28 | Taking a shower instead of a bath | very easy to do | somewhat easy to do | neither easy nor difficult | somewhat difficult to do | very difficult to do | not relevant for me / do not have that |  |
| 29 | Letting food cool down before setting it in the fridge or freezer | very easy to do | somewhat easy to do | neither easy nor difficult | somewhat difficult to do | very difficult to do | not relevant for me / do not have that |  |
| 30 | Putting in sealing tape in windows or doors where there is pull | very easy to do | somewhat easy to do | neither easy nor difficult | somewhat difficult to do | very difficult to do | not relevant for me / do not have that |  |
| 31 | Only heating the required amount of water when boiling water | very easy to do | somewhat easy to do | neither easy nor difficult | somewhat difficult to do | very difficult to do | not relevant for me / do not have that |  |
| 32 | Adjust the temperature of the fridge and freezer to the best efficiency | very easy to do | somewhat easy to do | neither easy nor difficult | somewhat difficult to do | very difficult to do | not relevant for me / do not have that |  |
| 33 | Taking short showers (about 3 minutes) | very easy to do | somewhat easy to do | neither easy nor difficult | somewhat difficult to do | very difficult to do | not relevant for me / do not have that |  |
| 34 | Adjusting the air condition temperature up one degree | very easy to do | somewhat easy to do | neither easy nor difficult | somewhat difficult to do | very difficult to do | not relevant for me / do not have that |  |
| 35 | Keeping the door of the warm oven or cold refrigerator closed as much as possible and only open it to take things in or out | very easy to do | somewhat easy to do | neither easy nor difficult | somewhat difficult to do | very difficult to do | not relevant for me / do not have that |  |
| 36 | Using energy saving LED lightbulbs everywhere | very easy to do | somewhat easy to do | neither easy nor difficult | somewhat difficult to do | very difficult to do | not relevant for me / do not have that |  |
| 37 | Remove the dust from the cooling coils of your fridge or freezer | very easy to do | somewhat easy to do | neither easy nor difficult | somewhat difficult to do | very difficult to do | not relevant for me / do not have that |  |
| 38 | Using lower temperatures for washing clothes | very easy to do | somewhat easy to do | neither easy nor difficult | somewhat difficult to do | very difficult to do | not relevant for me / do not have that |  |
| 39 | Heating water in a kettle instead of on the stove | very easy to do | somewhat easy to do | neither easy nor difficult | somewhat difficult to do | very difficult to do | not relevant for me / do not have that |  |
| 40 | Deice the fridge or freezer when it is icy | very easy to do | somewhat easy to do | neither easy nor difficult | somewhat difficult to do | very difficult to do | not relevant for me / do not have that |  |
| 41 | Avoiding using electricity during the peak hours | very easy to do | somewhat easy to do | neither easy nor difficult | somewhat difficult to do | very difficult to do | not relevant for me / do not have that |  |
| 42 | Switching appliances with standby completely off | very easy to do | somewhat easy to do | neither easy nor difficult | somewhat difficult to do | very difficult to do | not relevant for me / do not have that |  |
| 43 | Using thicker curtains during winter | very easy to do | somewhat easy to do | neither easy nor difficult | somewhat difficult to do | very difficult to do | not relevant for me / do not have that |  |
| 44 | Dishwasher: Using lower temperatures or shorter programs | very easy to do | somewhat easy to do | neither easy nor difficult | somewhat difficult to do | very difficult to do | not relevant for me / do not have that |  |
| 45 | Avoid using the tumble dryer | very easy to do | somewhat easy to do | neither easy nor difficult | somewhat difficult to do | very difficult to do | not relevant for me / do not have that |  |
| 46 | Turning down the heating with one degree | very easy to do | somewhat easy to do | neither easy nor difficult | somewhat difficult to do | very difficult to do | not relevant for me / do not have that |  |
| **Peak Hour Consumption** | | | | | | | | |
| 47 | Highest consumption during an hour last day |  |  |  |  |  |  |  |
| 48 | Which hour during that day did you consume the most? |  |  |  |  |  |  |  |
| **Intention to Save Electricity [3]** | | | | | | | | |
| 49 | I intend to save electricity during next week | very unlikely | somewhat unlikely | neither unlikely nor likely | somewhat likely | very likely |  |  |
| **Attitude to Saving Electricity [3]** | | | | | | | | |
| 50 | Saving electricity next week would be | very unpleasant | somewhat unpleasant | neither unpleasant nor pleasant | somewhat pleasant | very pleasant |  |  |
| **Perceived Behavioural Control to Save Electricity [3]** | | | | | | | | |
| 51 | I am confident that I am able to save electricity next week | totally false | somewhat false | neither false nor true | somewhat true | Totally true |  |  |
| 52 | Saving electricity next week is up to me | strongly disagree | moderately disagree | neither disagree nor agree | moderately agree | strongly agree |  |  |
| **Social Norms to Save Electricity [3]** | | | | | | | | |
| 53 | Most people who are important to me approve of me saving electricity next week | strongly disagree | moderately disagree | neither disagree nor agree | moderately agree | strongly agree |  |  |
| 54 | Most people like me save electricity next week | very unlikely | somewhat unlikely | neither unlikely nor likely | somewhat likely | very likely |  |  |
| **Habit Strength [4]** | | | | | | | | |
| 55 | Saving electricity is something I do without thinking | strongly disagree | moderately disagree | neither disagree nor agree | moderately agree | strongly agree |  |  |
| 56 | Saving electricity is something I do automatically | strongly disagree | moderately disagre | neither disagree nor agree | moderately agree | strongly agree |  |  |
| **Collective Efficacy [5]** | | | | | | | | |
| 67 | I think that we in [your country] are able to save electricity | strongly disagree | moderately disagree | neither disagree nor agree | moderately agree | strongly agree |  |  |
| 68 | I do not think that we in [your country] can make a difference with respect to saving electricity in the long run | strongly disagree | moderately disagree | neither disagree nor agree | moderately agree | strongly agree |  |  |
| **Emotional Reaction to Electricity Campaign** | | | | | | | | |
| 69 | How do you feel at the moment about your electricity consumption? | very negative | somewhat negative | neither positive nor negative | somewhat positive | very positive |  |  |
| 60 | I feel happy, when I think about my electricity consumption | strongly disagree | moderately disagree | neither disagree nor agree | moderately agree | strongly agree |  |  |
| 61 | I feel upset, when I think about my electricity consumption | strongly disagree | moderately disagree | neither disagree nor agree | moderately agree | strongly agree |  |  |
| 62 | I feel excited, when I think about my electricity consumption | strongly disagree | moderately disagree | neither disagree nor agree | moderately agree | strongly agree |  |  |
| **Reactance to Study (only last survey) [6]** | | | | | | | | |
| 63 | Did you feel participating in the project encroached on your freedom in any way? | not at all | a little bit | somewhat | quite a lot | very much so |  |  |
| **Self-reported Implementation of Energy Saving Behaviors** | | | | | | | | |
| 64 | reduced heating with at least one degree | no | yes | do not know |  |  |  |  |
| 65 | adjusted air conditioning up at least one degree | no | yes | do not know |  |  |  |  |
| 66 | shorter showers (about 3 min) | no | yes | do not know |  |  |  |  |
| 67 | Shower instead of bath | no | yes | do not know |  |  |  |  |
| 68 | Substituted less energy efficient light bulbs with LEDs | no | yes | do not know |  |  |  |  |
| 79 | I adjusted the temperature of the fridge or freezer to a more efficient level | no | yes | do not know |  |  |  |  |
| 70 | I deiced the fridge or freezer | no | yes | do not know |  |  |  |  |
| 71 | I removed dust from the cooling coils of the fridge or freezer | no | yes | do not know |  |  |  |  |
| 72 | I let warm food cool down outside the fridge or freezer first | no | yes | do not know |  |  |  |  |
| 73 | I reduced the temperature for most of the washing in my washing machine | no | yes | do not know |  |  |  |  |
| 74 | I used lower temperatures or shorter programs in my dishwasher | no | yes | do not know |  |  |  |  |
| 75 | I did not use my tumble dryer to dry clothes | no | yes | do not know |  |  |  |  |
| 76 | I used a kettle to warm water | no | yes | do not know |  |  |  |  |
| 77 | I only heated the required amount of water when cooking | no | yes | do not know |  |  |  |  |
| 78 | I remembered to keep the lids on the pots when cooking | no | yes | do not know |  |  |  |  |
| 79 | I did not open the door of the oven or fridge unnecessarily | no | yes | do not know |  |  |  |  |
| 80 | I switched devices with standby completely off | no | yes | do not know |  |  |  |  |
| 81 | I tried to reduce my electricity consumption in the peak hours | no | yes | do not know |  |  |  |  |
| 82 | I have put up thicker curtains | no | yes | do not know |  |  |  |  |
| 83 | I have put in sealing tape where it pulled | no | yes | do not know |  |  |  |  |

*Note.* The initial survey included the first 46 items, while the remaining items were measured repeatedly over the course of 6 weeks at 6 different time points.

**References**

1. Reichl J, Cohen JJ, Klöckner CA, Kollmann A, Azarova V. The drivers of individual climate actions in Europe. Global Environmental Change. 2021;71:102390.

2. Schultz PW. The structure of environmental concern: Concern for self, other people, and the biosphere. Journal of environmental psychology. 2001;21(4):327-39.

3. Klöckner CA. A comprehensive model of the psychology of environmental behaviour—A meta-analysis. Global environmental change. 2013;23(5):1028-38.

4. Verplanken B, Orbell S. Reflections on past behavior: a self‐report index of habit strength 1. Journal of applied social psychology. 2003;33(6):1313-30.

5. Zaccaro SJ, Blair V, Peterson C, Zazanis M. Collective efficacy. Self-efficacy, adaptation, and adjustment: Theory, research, and application. 1995:305-28.

6. Hong S-M, Page S. A psychological reactance scale: Development, factor structure and reliability. Psychological Reports. 1989;64(3_suppl):1323-6.
