# Supplementary material for: Promoting electricity conservation through behavior change: A study protocol for a web-based multiple-arm parallel randomized controlled trial
Source: PLoS One. 2024 Mar 14;19(3):e0293683. doi: 10.1371/journal.pone.0293683 (PMC10939288; doi:10.1371/journal.pone.0293683)
Supplement: S3 Table — (DOCX) [file pone.0293683.s003.docx]

**S3 Table.** *Independent and dependent variables, socio-demographic and psychological covariates, and moderator confounders*

| **Independent variables:** | **Dependent variables** | **Covariate/moderator variables** | |
| --- | --- | --- | --- |
| ***Experimental treatment:*** | 1. the weekly electricity consumption (electricity meter readings or the weekly consumption data) | ***Socio-demographics:*** | ***Psychological variables:*** |
| 1. control 1 (weekly measurement but no interventions) |  | 1. country of residence | 1. identification with the country one lives in |
|  |  | 2. region within the country | 2. climate change worry |
| 2. control 2 (only measurement at the start and at the end of the six weeks) |  | 3. age | 3. environmental concern |
|  | 2. implementation of electricity saving behaviors on a weekly basis | 4. gender | 4. concern about using too much electricity |
| 3. information |  | 5. number of people in the household | 5. personal norms |
| 4. information + social norm |  | 6. number of children under 6 | 6. perceived difficulty of implementing the different energy tips |
| 5. information + collective framing | 3. the hour during the day before the meter reading the peak load was measured and how high this peak was in kWh (only in Norway) | 7. number of children 6-11 |  |
| 6. information + social norm + collective framing |  | 8. number of children 12-17 | 7. intention to save electricity |
| 7. information + commitment |  | 9. education level | 8. attitude to saving electricity |
| 8. information + feedback |  | 10. job situation | 9. perceived behavioral control to save electricity |
| 9. information + feedback + collective framing |  | 11. social status | 10. social norms to save electricity |
| 10. information + feedback + competition |  | 12. risk of energy poverty and energy related technical situation in the household | 11. electricity saving habits |
| 11. information + feedback + competition + collective framing |  | 13. energy saving lightbulbs | 12. collective efficacy |
|  |  | 14. efficient washing machine | 13. emotional reaction to electricity campaign |
| 12. information + social norm + commitment |  | 15. efficient dishwasher | 14. reactance |
|  |  | 16. heat pump | ***Covariates from other sources:*** |
| 13. information + social norm + commitment + feedback + competition |  | 17. tumble dryer | 1. average electricity prices on a weekly level |
|  |  | 18. air condition | 2. average temperatures on a weekly level |
|  |  | 19. charging an electric car at home |  |
| 14. information + social norm + commitment + feedback + competition + collective framing info + social |  | 20. heating primarily with electricity | 3. predominant electricity tariff type in the region the household is in |
|  |  | 21. producing warm water primarily with electricity |  |
