# Supplementary material for: Promoting electricity conservation through behavior change: A study protocol for a web-based multiple-arm parallel randomized controlled trial
Source: PLoS One. 2024 Mar 14;19(3):e0293683. doi: 10.1371/journal.pone.0293683 (PMC10939288; doi:10.1371/journal.pone.0293683)
Supplement: S1 File — (DOCX) [file pone.0293683.s005.docx]

**S7 File.** *Data Management and Confidentiality*

In the ENCHANT project, ensuring the personal privacy of participants is a top priority. During the campaign, only email addresses are collected for effective communication, stored separated from the other data, and protected in accordance with data protection regulations. Access to relevant data is restricted to the NTNU research team and our software provider, NRGsurf, ensuring stringent confidentiality. After the campaign, data will be anonymized and, with participants’ explicit consent, used for analysis and published as reports and academic articles. The anonymized dataset will be openly accessible, with all personal information removed to ensure individual identities are safeguarded. Participants’ consent to participate serves as the legal basis for processing their personal data, which has been approved for compliance with data protection legislation by the Norwegian Agency for Shared Services in Education and Research (SIKT, formerly NSD; case number 120694).
