# Supplementary material for: Promoting electricity conservation through behavior change: A study protocol for a web-based multiple-arm parallel randomized controlled trial
Source: PLoS One. 2024 Mar 14;19(3):e0293683. doi: 10.1371/journal.pone.0293683 (PMC10939288; doi:10.1371/journal.pone.0293683)
Supplement: S2 File — (DOCX) [file pone.0293683.s006.docx]

## S8 File. *Are you interested in taking part in the research project "Energy Efficiency in Norwegian Households"?*

This is an inquiry about participation in the ENCHANT research project funded by European Union’s Horizon 2020 research and innovation programme (project number 957115). The project aims to investigate measures for increasing energy efficiency in European households. In this letter, we will give you information about the purpose of the project and what your participation on our intervention platform will involve.

### Purpose of the project

The research project “Energy Efficiency through behaviour Change Transition Strategies (ENCHANT)” tests established tools to inspire energy saving in real-life conditions. These tools have been developed, fitted, and are now tested to help households to save electricity (and thereby also costs and emissions) in different regions in Europe in collaboration with local user partners.

For this purpose, ENCHANT’s research team in Norway collaborates with Viken county and Naturvernforbundet to test how different types and combinations of these tools work. Therefore, we have programmed the electricity saving platform that you are now on. Through the platform, we ask you to report your weekly electricity use for six weeks and answer a short questionnaire once a week which helps us understand your motivations and how they change. We will send you useful information about your electricity consumption and tips for how you can reduce it and save money (at the same time releasing pressure from the energy system).

### Who is responsible for the research project?

The Department of Psychology at the Norwegian University of Science and Technology (NTNU) is responsible for conducting the project’s survey and the data processing. The software company NRG surf (https://www.nrgsurf.de/en/) has programmed the platform and assists in processing of the non-personal data. Viken county and Naturvernforbundet are responsible for spreading the invitation to participate in the project through their communication channels but do not have access to the data.

### Why are you being asked to participate?

You are invited to participate in the ENCHANT saving platform because you are living in Viken county, are a member of Naturvernforbundet, or have been following their communication campaigns. If you are 18 years or older, you can participate. It is not necessary, that you live in Viken or are connected to Naturvernforbundet. The only restriction is that you need to live in Norway.

### What does participation involve for you?

The participation in this electricity saving campaign and research project means that you first answer a short online questionnaire which takes about 5-10 minutes. Starting a couple of days later, we will ask you once a week for six weeks to read your electricity meter and report the number you find there. If you have access to your hourly consumption data through your electricity net provider (either on their website or through an app that most energy net providers offer for download), we also ask you report the hour on one of the days last week with the highest electricity consumption and how much that was to get an idea, when during the day you use most energy. Furthermore, we ask you to answer a very short questionnaire once a week (3-5 minutes). We will randomly place participants into different groups during the campaign. Depending on which group you are in, we send you different types of information about your electricity use, and we will give you tips for how you can reduce your electricity use and energy bill. If you miss a weekly survey, we will send you one reminder.

### Participation is voluntary

Participation in the project is voluntary. If you chose to participate you can withdraw your consent at any time without giving a reason. In the electricity saving platform, you can end your participation in the campaign, delete all data we collected from you, and/or delete your user account. There will be no negative consequences for you if you chose not to participate or later decide to withdraw.

### Your personal privacy – how we will store and use your personal data

To be able to communicate with you during the campaign, we need your e-mail address (please use an e-mail address you check frequently). This is the only identifying information we are going to collect from you. The mail addresses are stored on a different server environment separated from the consumption and survey data. No other identifying data will be recorded in the project.

We will only use your personal data for the purpose(s) specified in this information letter. The data is collected for research within the ENCHANT project, which includes publication in scientific journals, presentations on conferences, and policy advice. Only anonymised data will be used for this purpose. Only the research team at NTNU has access to the mail addresses (also the software company does not have access to the mail server). All data is stored and processed on Servers provided by Digital Oceans in the Netherlands, following European data protection regulations.

We will process your personal data confidentially and in accordance with data protection legislation (the General Data Protection Regulation and Personal Data Act).

The NTNU research team is responsible for gathering and processing the data.

Only the NTNU research team will have access to the personal data (e-mail addresses).

NRG surf has developed the software for data collection. They have access to the survey and electricity use data, but not the e-mail addresses. By contract, they are bound to follow strict data protection rules and treatment of the data in line with GDPR.

No unauthorized persons can access the personal data.

After the data collection is ended, the data material will be encrypted and stored on NTNU's server

Data that can be directly linked to individual participants (e-mail adresses) will be stored separately from rest of the data and deleted as early after the data collection as possible

ENCHANT’s project partners have access to the anonymised data, which will be shared by the NTNU research team.

Data that can be directly linked to individual participants will NOT be shared with project partners and others.

With your permission, data from the survey will be used for a detailed analysis. The results of the study will be published in the form of reports and academic articles. The anonymized dataset will be made available open access after the end of the project in line with the open data regulations of the EU. All personal information you provide is considered completely confidential. Your e-mail address or name will be removed from the final dataset and will not appear in any thesis or report resulting from this survey.

### What will happen to your personal data at the end of the research project?

The ENCHANT project is scheduled to end 31.12.2023. The data collected in this campaign, however, will be anonymised at the earliest point of time (i.e., latest 31.10.2023) so that the answers cannot be traced back to the individual.

### Your rights

As long as you can be identified in the collected data, you have the right to:

- request that your personal data is deleted (this can be done in the platform)
- access the personal data that is being processed about you
- request that incorrect personal data about you is corrected/rectified
- receive a copy of your personal data (data portability), and
- send a complaint to the Data Protection Officer or The Norwegian Data Protection Authority regarding the processing of your personal data

### What gives us the right to process your personal data?

We will process your personal data based on your consent. Registering for the campaign after you have read this information is considered as consent to participate. Based on an agreement with the Department of Psychology, NTNU,NSD – The Norwegian Centre for Research Data AS has assessed that the processing of personal data in this project is in accordance with data protection legislation.

### Where can I find out more?

You can find more information here [GDPR](https://app.enchant-project.eu/article/gdpr)

If you have questions about the project or want to exercise your rights, contact:

- Project leader: Prof. Christian A. Klöckner, Department of Psychology, NTNU, by email: ([enchant@ips.ntnu.no](mailto:enchant@ips.ntnu.no)) or by telephone: +47 73 59 19 77.
- Data Protection Officer: Thomas Helgesen, Director Organization, NTNU, by email: ([thomas.helgesen@ntnu.no](mailto:thomas.helgesen@ntnu.no))or by telephone: +47 930 79 038.
- NSD – The Norwegian Centre for Research Data AS, by email: ([personverntjenester@nsd.no](mailto:personverntjenester@nsd.no)) or by telephone: +47 55 58 21 17.
