# Supplementary material for: Promoting electricity conservation through behavior change: A study protocol for a web-based multiple-arm parallel randomized controlled trial
Source: PLoS One. 2024 Mar 14;19(3):e0293683. doi: 10.1371/journal.pone.0293683 (PMC10939288; doi:10.1371/journal.pone.0293683)
Supplement: S3 File — (PDF) [file pone.0293683.s007.pdf]

Notification form

Reference number

120694

What personal data will you process?

- Name
- Web identifier

Project information

Project title: ENCHANT - Electricity saving intervention and survey platform

Executive Summary

ENCHANT - Energy Efficiency through behaviour Change Transition - is a project that aims to support the energy transition by testing the impact of interventions affecting energy consumption behaviour on a large-scale across Europe. The interventions will be developed, fitted, and tested with the objective to unlock an energy efficiency potential in the public, through behavioural change. This application is for the standardised intervention platform that will role-out interventions in a randomised control trial across six European countries (Norway, Austria, Turkiye, Germany, Romania, and Italy).

Justify the need to process the personal data

Personal data (e-mail address) is required to be able to communicate with the participants in this longitudinal intervention study.

Project description -> 00_ENCHANT_platform_description.pdf

External funding

- Public authorities

Type of project

- Research/doctoral project

Responsibility for processing

Institution responsible for processing: Norwegian University of Science and Technology / Faculty of Social and Educational Sciences (SU) / Department of Psychology

Project manager

Christian Klöckner, christian.klockner@ntnu.no, tel: +4773591977

Is the responsibility for processing shared with several institutions?

No

Sample 1

Describe the sample

The sample recruited to this study are household representatives (general population), 18 years or older in Norway, Germany, Austria, Turkiye, Romania, and Italy.

Describe how to find or contact the sample

The participants are recruited by the user partners in the ENCHANT consortium. They promote the campaign through their communication channels (websites, posters, newsletters, facebook, twitter, newspaper advertisement, etc.). User partners engaged in ENCHANT are: - Viken fylkeskommune, administration (NO) - Naturvernforbundet, NGO (NO) - Badenova, energy provider (DE) - Clusj Napoca Municipality, administration (RO) - Electrica, energy provider (RO) - Izmir metropolitan municipality, administration (TR) - Gediz, energy provider (TR) - EnergieKompass, energy community (AT) - Energia Positiva, energy provider (IT) - Fonfazione Roffredo Caetani/Ninfa gardens, NGO (IT) The user partners direct the participants to the platform landing page, where they can register for a campaign in their respective country after having read the project information and informed consent sheet.

Age group

18 - 120

What personal data will be processed about the sample? 1

- Name
- Net identifier

How is the information about sample 1 collected?

- Electronic questionnaire

Attachment -> 03_Initial_survey.pdf

Legal basis for processing general personal data

Consent (General Data Protection Regulation Article 6(1)(a))

Electronic questionnaire

Attachments -> 08_Repeated_survey_first_week.pdf

Lawful basis for processing general personal data

Consent (Article 6(1)(a) of the General Data Protection Regulation)

Electronic questionnaire

Attachments -> 08_Repeated_survey_week_2-5.pdf

Lawful basis for processing general personal data

Consent (Article 6(1)(a) of the General Data Protection Regulation)

Electronic questionnaire

Attachments -> 08_Repeated_survey_last_week.pdf

Lawful basis for processing general personal data

Consent (Article 6(1)(a) of the General Data Protection Regulation)

Electronic questionnaire

Attachments -> 06_a_Welcome_screen_smart_meter.pdf

Lawful basis for processing general personal data

Consent (Article 6(1)(a) of the General Data Protection Regulation)

Electronic questionnaire

Attachments -> 07_Weekly_electricity_measurement.pdf

Lawful basis for processing general personal data

Consent (Article 6(1)(a) of the General Data Protection Regulation)

Information to sample 1

Does the committee receive information about the processing of personal data?

Yes

How does the committee receive information about the processing?

In writing (paper or electronic)

Information letter -> 01_Informed_Consent.pdf

Third parties

Does the project collect information about third parties?

No, we do not

Documentation of consent

How are consents documented?

Electronically (e-mail, e-form, digital signature)

How can consent be withdrawn?

The survey platform allows the participants to self-administer their campaign participation (they can stop at any point and will then not be contacted anymore), they can also delete their account, by which all of their data in the system will be deleted. Furthermore, they can contact the responsible person for the study to ask for manual removal of their data (see consent form).

How can data subjects access, correct or delete their personal data?

The participants can contact the main responsible person for the study and request to get a copy of all data stored about them.

Total number of data subjects in the project

10.000-49.999

Authorisations

Will any of the following approvals or authorisations be obtained?

Other authorisation

Other authorisation

The platform design has been discussed in detail with the data protection officer at NTNU. As a result, we changed the architecture of the platform so that the server that stores personally identifying data (names, email addresses) is now located in NTNU's infrastructure, while only non-identifying data is stored on the Digital Ocean server. Nevertheless, a data processing agreement was signed with Digital Ocean that also contains an SCC.

Authorisations -> Uttalselse datavernombudet NTNU.pdf

Security measures

Will personal data be stored separately from other data?

Yes, it will

What technical and physical measures will secure the personal data?

Encrypted transmission

Encrypted storage

Access restriction

Access log

Continuous anonymisation

Multi-factor authentication

Where is the personal data processed?

Data processor

Hardware

Who has access to the personal data?

Project manager

Data processor

Others with access to the personal data

Which data processor has access to the personal data?

NRGsurf: Programmed the platform and will assist in technical question occurring during the campaign. They have no access to personal data (names and e-mails are stored in a password protected NTNU server which they will not have the password to from when the campaign starts). NRGsurf signed a contract that binds them to GDPR regulations. NTNU: NTNU operates the server that stores personal information (names, e-mails). During the setup of the server, NGRsurf has access to secure that it operates as planned. After that their user accounts will be deleted before personal

data of participants is stored there. From here on, only NTNU researchers have access. DigitalOcean: The campaign platform is hosted on servers provided by DigitalOcean. The servers are located in Amsterdam (within the EU) and are operated according to European data protection regulations. A data processing agreement with DigitalOcean is in place regulating that they are obliged to follow GDPR and other European data protection regulations. Part of that data processing agreement are Standard Contractual Clauses (SCC) which regulates the handling of data which is processed by an entity which is owned by a US company. Only deidentified data is stored on this server. Cloudflare: We use cloudflare to secure security and privacy of the platform. The use of Cloudflare is based on our legitimate interest in a provision of our website offerings that is as error free and secure as possible (Art. 6(1)(f) GDPR). Data transmission to the US is based on the Standard Contractual Clauses (SCC) of the European Commission. Details can be found here: https://www.cloudflare.com/privacypolicy/. A data processing agreement with DigitalOcean is in place regulating that they are obliged to follow GDPR and other European data protection regulations. Sendinblue: We use sendinblue as a solution to send out the mails from our system. Sendinblue is located in Germany, underlying the European data protection regulations. A data processing agreement with DigitalOcean is in place regulating that they are obliged to follow GDPR and other European data protection regulations.

Who else has access to the personal data?

NTNU was responsible for developing the platform and all data collection design. ENCHANT scientific partners (NTNU [no], Roma3 university [it], Babes-Bolyai University [ro], energy institute [at], Smart Innovation Norway [no], NTNU social research [no], Izmir Economic University [tr]): The analyses of the data will be conducted in the consortium under the lead of NTNU, Trondheim, Norway, and the Energy institute of the Johannes Keppler University in Linz, Austria. The other scientific partners are involved to a smaller degree. All analysis will be conducted based on unidentified datasets (participants are represented by codes). No personal data will be shared with the partners. All ENCHANT partners are bound by the Grant Agreement and the Consortium Agreement to follow the processes in the Data Management Plan of the project, which secures GDPR conformity of all activities.

Is personal data transferred to a third country?

Yes

Transfers/disclosure of data outside the EU/EEA

Digital Ocean, USA, Art. 46 with additional measures

Conclusion

Project period

02.01.2023 - 31.12.2023

What happens to the data at the end of the project?

Data is anonymised (deletes/rewrites the personal data)What anonymisation measures will be taken?

The link key will be deleted

Personally identifiable information will be removed, rewritten or roughly categorised

Will individuals be recognisable in the publication?

No, no

Additional information

The project description shows the project design and all screenshots and info in detail. I upload everything here to have things in an orderly fashion. NTNU was the sole responsible for designing the study and the platform (with technical help by NRGsurf), analyses will be conducted on the pseudonymised/anonymised data only, here the other consortium partners are also involved. We therefore do not consider them "behandlingsansvarlige", but they are legally bound by grant agreement and consortium agreement to follow the Data Management Plan of the project (also attached). The technical design has now been discussed and approved by NTNU's data protection officer (see attached assessment). We have conducted a risk assessment of the following aspects: Data breach scenario: We assess that a data breach might happen at two places in the data processing, with the part of the data that is hosted on Digital Ocean's servers, and at NTNU's servers. The impact of the first type of data breach would be minor as none of the data is identifiable and there will be no irreversible effects of a data breach. The likelihood is low, as there are no documented cases of a breach of data stored on digital ocean servers in the last year. There has been a breach of customer billing data, though in April 2022. A data breach of the NTNU servers might in the worst case expose the participants' names and e-mail addresses and the password for the platform. Participants are instructed to choose a password specifically for this website and not reuse it elsewhere. In case of such a breach, participant will be informed and asked to change their user data. A leakage of e-mail addresses might lead to being exposed unwanted advertisement or phishing attempts. In case of a data breach, participants will be instructed to carefully evaluate unexpected contacting. During the last year, no breach of personal data has happened at NTNU. Therefore the risk is evaluated as low with manageable consequences. Inability to exercise rights scenario: Participant will give informed consent as outlined above, which includes an easily understandable instruction for how to exercise their rights. Therefore, we see the likelihood of an inability to exercise rights scenario as low. The impact of this scenario is assessed as being minor, as there will be no irreversible negative effects (e.g., people can just stop answering a survey, the survey data is non-sensitive and there are no impacts in people's lives if the collected data is incorrect). Intrusion of secluded spaces scenario: Not relevant for this project. Inappropriate outcomes to people: Not relevant for this project.

Other attachments

ENCHANT_D1.5_DMP_R1.pdf

00_Complete_Platform_Documentation.pdf

00_ENCHANT_platform_description.pdf

Uttalselse datavernombudet NTNU.pdf
